# V-ATPase-targeted siRNA library screening reveals ATP6V1A negatively regulates UVB-induced keratinocyte senescence

**DOI:** 10.1186/s13062-026-00891-4

**Published:** 2026-07-15

**Authors:** Peiqi Lian, Xuyi Deng, Qingqiu Wen, Yinyun Deng, Fenghao Liu, Hui Liu, Zhenhua Ding, Meijuan Zhou, Yinghui Wang

**Affiliations:** Department of Radiation Medicine, Guangdong Provincial Key Laboratory of Tropical Disease Research, School of Public Health, Southern Medical University, Guangzhou, 510515 China

**Keywords:** UVB, Photoaging, siRNA library, Lysosome, ATP6V1A

## Abstract

Photoaging is a form of premature skin aging mainly induced by long-term exposure to ultraviolet exposure. Lysosomes are key organelles responsible for the degradation and recycling of intracellular components and are essential for maintaining metabolic and nutrient homeostasis. Although lysosomal dysfunction is closely associated with cellular aging, the role of V-ATPase in regulating lysosomal function during photoaging remains incompletely understood. By screening a V-ATPase-targeted siRNA library and validating the results using publicly available single-cell transcriptomic datasets, we identified ATP6V1A as a key regulator of UVB-induced cellular senescence. Furthermore, ATP6V1A knockdown exacerbated the UVB-induced cellular senescence and impaired lysosomal acidification and membrane integrity, whereas ATP6V1A overexpression effectively alleviated keratinocyte senescence, lysosomal dysfunction and autophagy inhibition. Moreover, treatment with the V-ATPase inhibitor BafA1 aggregated cellular senescence phenotype and autophagy inhibition and this phenomenon partially reversed by ATP6V1A overexpression. Collectively, ATP6V1A promotes autophagy by regulating lysosomal function, thereby relieving UVB-induced cellular senescence.

**Supplementary Information:**

The online version contains supplementary material available at https://doi.org/10.1186/s13062-026-00891-4.

## Introduction

Cellular senescence is a state of permanent growth arrest, characterized by the upregulation of cyclin-dependent kinase inhibitors, enhanced anti-apoptotic resistance, increased senescence-associated β-galactosidase (SA-β-gal) activity, and the emergence of the senescence-associated secretory phenotype [1, 2]. This cellular state can be triggered by endogenous and exogenous stresses and is involved in various age-related pathologies. As the most externally exposed organ, the skin is highly susceptible to environmental insults, particularly ultraviolet (UV) radiation, which is the primary factor causing skin photoaging [3, 4]. UV radiation induces permanent cell cycle arrest and ultimately photoaging through the generation of various DNA lesions, such as base damage, single-strand breaks, and double-strand breaks [5]. Furthermore, the accumulation of reactive oxygen species induced by UV radiation contributes to photoaging [6, 7]. However, the precise mechanisms underlying UVB-induced photoaging remain to be elucidated.

Lysosomes are widely recognized as the primary organelle responsible for the degradation and recycling of cellular components, processes that are essential for maintaining metabolic and nutrient homeostasis [8]. Therefore, growing evidence suggests that lysosomal dysfunction is closely linked to cellular senescence and tissue aging. Incomplete degradation of lipofuscin accumulated in lysosomes is a hallmark of aging [9]. Photoaged fibroblasts showed impaired lysosomal degradation capacity, characterized by reduced Cathepsin D (CTSD) expression and decreased lysosomal acidity, a key feature of aging cells [10–12]. The vacuolar H⁺-ATPase (V-ATPase), a proton pump complex embedded in the lysosomal membrane, is essential for maintaining the acidic intraluminal pH required to support the optimal activity of diverse lysosomal enzymes [13]. Mutations in V-ATPase subunits have been associated with multiple age-related diseases, including osteoporosis and Parkinson’s disease [14, 15]. Pharmacological enhancement of V-ATPase assembly restores lysosome-dependent autophagy and thereby alleviates replicative senescence [16]. However, the role of V-ATPase in regulating lysosomal function during UVB-induced cellular senescence has yet to be fully elucidated.

In this study, we observed lysosomal dysfunction in UVB-induced cellular senescence. By integrating single-cell data from aged cells with V-ATPase siRNA library screening, we identified the V-ATPase subunit ATP6V1A as a key gene in the UVB-induced skin senescence process. Genetic knockdown of ATP6V1A exacerbated both the UVB-induced cellular senescence phenotype and lysosomal damage. In addition, pharmacological inhibition of V-ATPase by bafilomycin A1 (BafA1) promotes UVB-induced cellular senescence phenotype, which was reversed by ATP6V1A overexpression. Collectively, our findings establish ATP6V1A as a key regulator of UVB-induced senescence through modulating lysosomal function, highlighting its potential as a therapeutic target for intervention.

## Materials and methods

### Cell culture

The HaCaT cell line was purchased from Shanghai HonSun Biotechnology Co., Ltd. and maintained in Dulbecco’s Modified Eagle Medium (Gibco, USA), supplemented with 10% fetal bovine serum (Everygreen, China), 100 µg/mL streptomycin, and 100 U/mL penicillin (Beyotime China). Cells were cultured at 37℃ in a humidified 5% CO_2_ environment.

### UVB radiation induced senescence in HaCaT cells

HaCaT cells were irradiated with UVB using a Philips lamp at a dose of 20 J/m^2^, twice daily at 12 h intervals for 4 consecutive days. Prior to each irradiation, the culture medium was removed and the cells were gently washed with PBS. To ensure consistent dosing, the minimum volume of PBS required to fully cover the cell layer was used, with 1.8 mL for 60-mm culture dishes and volumes adjusted proportionally to the growth surface area for other culture vessels. The non-irradiated control group underwent the same procedures but without UVB irradiation.

### UVB radiation induced senescent skin in mice

The animal experiments were approved by the Animal Care and Use Committee of Southern Medical University (Approval No. SMUL202509022). Male BALB/c mice aged 4–6 weeks were purchased from the Guangdong Provincial Medical Laboratory Animal Center, randomly assigned to two groups, and acclimatized. Dorsal hair was shaped approximately 1 × 2 cm area. After 24 h, mice were anesthetized and subjected to UVB irradiation. was under according to the protocol [3]: Week 1: 0.1 MED (60 mJ/cm^2^); Week 2: 0.25 MED; Week 3: 0.5 MED; thereafter, 0.5 MED was administered 5 days per week until a cumulative dose of 12 MED was reached. After irradiation, the mice were euthanized, and irradiated skin samples were collected. After fixing the tissue with 4% paraformaldehyde, it was embedded in paraffin, sectioned, and then used for subsequent experiments.

### Cell transfection

Cells were seeded in 60 mm culture dishes and cultured for 24 h until reaching 50–60% confluence. Transfection was performed according to the protocol for Lipofectamine™ 2000 (Invitrogen, USA) transfection reagent. Overexpression was achieved using 2 µg of ATP6V1A plasmid or empty vector (Ribo, China). Knockdown was performed using ATP6V1A siRNA or siNC (Ribo, China). Medium was replaced after 6 h.

### Single-cell RNA sequencing data analysis and aging score calculation

To identify the potential biological functions and molecular pathways involved in skin aging, the raw scRNA-seq data were retrieved from the Gene Expression Omnibus (GEO) database under accession number GSE130973. Differentially expressed genes (DEGs) were identified using the Seurat R package, and Gene Ontology (GO) enrichment analysis was subsequently performed using the clusterProfiler R package to screen for significantly enriched biological processes. To further evaluate the association between V-ATPase-related genes and aging, aging scores were calculated using the AUCell R package. Aging genes were defined based on the classification by Yu et al. [17]. The entire cell population was divided into two groups, “OLD_High” (*n* = 1,027) and “OLD_Low” (*n* = 1,044), based on the median AUCell score for V-ATPase. Statistical comparison of the AgingScore between the two groups was performed and visualized using the ggbetweenstats function from the ggstatsplot R package.

### siRNA library screening

HaCaT cells were seeded at 10,000 cells per well in 48 well plates. After attachment, cells were transfected with the ATPase siRNA library and exposed to UVB irradiation for 4 days. After irradiation, remove the cell culture medium and wash once with PBS, following the manufacturer’s instructions (SG-03, Dojindo, Japan). Next, prepare a 1:1000 working solution of Bafilomycin A1 (BafA1) and add it to the culture wells, then incubate for 1 h in a 37 °C, 5% CO_2_ incubator. Next, a 1:1000 working solution of SPiDER-βgal was prepared and added to the dish, followed by incubation under the same conditions for 30 min. After incubation, the supernatant was discarded, and the cells were washed twice with PBS. The cells were digested with trypsin and resuspended in PBS. Analyze the cells using a flow cytometer and normalize cell counts using CCK-8 as a reference. Significance for each gene was evaluated using the *Z*-factor, calculated as $\:Z=1-\frac{3{\sigma\:}_{s}+3{\sigma\:}_{c}}{|\:{\mu\:}_{s}-{\mu\:}_{c}|}$, where *σ*_*s*_ is the mean fluorescence intensity after single gene knockout, *σ*_*c*_ is the mean fluorescence intensity of the control, *µ*_*s*_ is the standard deviation of fluorescence intensity after single gene knockout, and *µ*_*c*_ is the standard deviation of fluorescence intensity in the control.

### Western blot

HaCaT cells were seeded in 60 mm culture dishes and cultured for 24 h prior to UVB irradiation. Cells were harvested 12 h post-irradiation for protein extraction. Total protein concentration was determined using a BCA assay kit (Beyotime, China). Equal amounts of denatured protein samples were separated by SDS-PAGE and subsequently electroblotted onto 0.45 μm PVDF membranes. The membranes were blocked with 5% non-fat milk (diluted in TBST) for 1 h at room temperature and then incubated with primary antibodies overnight at 4℃. Primary antibodies and their dilutions were as follows: ATP6V1A (1:10,000, Proteintech 17115-1-AP, China), LC3B (1:2,000, Merck L7543, USA), SQSTM1/p62 (1:2,000, Proteintech 29503-1-AP, China), HA (1:5000, Proteintech 51064-2-AP, China), and β-actin (1:10000, 66009-1-Ig, Proteintech, China). After washing with TBST, membranes were incubated at room temperature with either HRP-labeled goat anti-mouse IgG (H + L) (1:4000, Beyotime A0216, China) or HRP-labeled goat anti-rabbit IgG (H + L) (1:4000, Beyotime A0218, China). Protein bands were visualized using an enhanced chemiluminescence reagent and imaged with a Tanon imaging system (Tanon, China). Finally, bands were quantitatively analyzed using ImageJ software.

### Immunofluorescence (IF)

Cells were seeded at a density of 6 × 10^4^ cells per well in a 24 well plate. After 24 h of culture, fix the cells with ice-cold methanol and block them at room temperature for 10 min with 5% goat serum and 0.3% Triton™ X-100 (diluted in PBS). Subsequently, cells were incubated with the corresponding primary antibody. Antibodies and dilution ratios used: γH2A.X (1:250, CST 9781, USA), p16 (1:50, Santa Cruz Biotechnology, sc-1661 USA), LAMP1 (1:250, Santa Cruz Biotechnology, sc-20011, USA), Galectin-3 (1:200, CST 87985, USA). Incubated overnight at 4℃. The following day, after two washes with PBS, the slides were incubated with the corresponding fluorescent secondary antibodies at room temperature for 1 h. Antibodies and dilution ratios used: CoraLite488-conjugated Goat Anti-Mouse IgG (H + L) (1:250, Proteintech SA-00013-1, China), Multi-rAb™ CoraLite^®^ Plus 594-Goat Anti-Rabbit Recombinant Secondary Antibody (H + L) (1:250, Proteintech RGAR004, China). Finally, slides were mounted using ProLong Gold anti-fluorescence quenching mounting medium containing DAPI, and fluorescence images were acquired using a confocal microscope (Zeiss, Germany). Immunofluorescence intensity for cells and tissue sections was quantified by analyzing nine distinct fields of view using ImageJ software. The integrated density of the target signal was normalized to DAPI fluorescence to account for density and area differences. All data were subsequently normalized to the control group and presented as relative fluorescence density. Quantitative colocalization analysis using Pearson’s correlation coefficient with the Coloc 2 plugin in ImageJ.

### SA-β-gal staining

SA-β-gal staining was used the SA-β-gal assay kit (Beyotime, China). Following UVB irradiation, block cells with blocking solution for 30 min according to the instructions. After blocking, wash three times with PBS for 5 min each. Add the pre-prepared staining solution and incubate at 37 °C in a CO_2_-free environment for 24 h. Discard the staining solution, replace with PBS, and image under the optical microscope (Zeiss, Germany). Randomly select 5 fields of view per well. Count the blue cells and total cells within each field of view, then calculate the positive rate by determining the proportion of blue cells relative to the total cell count. The positivity rate per well is the average of the 5 fields of view. For the quantification of tissue SA-β-gal positivity, ImageJ was utilized to measure both the epidermal area and the SA-β-gal-positive area. The positivity rate was defined as the proportion of the positive area relative to the total epidermal area.

### LysoSensor staining

Quantify changes in lysosomal acidity using the LysoSensor Kit (Yeasen, China). Following the kit instructions, 1 mM LysoSensor was added to pre-warmed DMEM medium at 37℃ at a 1:1000 dilution, yielding a working solution concentration of 1 µM. The cells were incubated at 37℃ in 5% CO_2_ for 30 min. After incubation, the working solution was discarded, replaced with PBS, and images were captured using a fluorescence microscope (Zeiss, Germany). The method for quantifying the fluorescence intensity of LysoSensor is the same as above.

### eRFP-EGFP-LC3 lentivirus infection and autophagic flux evaluation

HaCaT cells were seeded at a density of 2 × 10^5^ cells per well in 35 mm culture dishes. After 24 h of culture, the medium was replaced with fresh medium containing an appropriate amount of eRFP-EGFP-LC3 lentivirus (Hanheng, China; multiple infections, MOI = 100, viral titer 1 × 10^8^ TU/mL). To enhance transduction efficiency, the polycationic agent Polybrene (final concentration 8 µg/mL) was added concurrently. Subsequently, positive cells were selected using 0.5 µg/mL puromycin and passaged at least three times until all cells stably expressed the target protein. Cells stably infected with eRFP-EGFP-LC3 lentivirus were replated into 24 well plates and cultured for an additional 24 h.

To assess autophagy flux, cells were subjected to UVB irradiation following knockdown or induction of ATP6V1A overexpression, as well as treatment with BafA1. Following irradiation, cells were fixed with ice-cold methanol and imaged using a confocal microscope (Carl Zeiss, Germany). Autophagosomes appeared as yellow fluorescent spots (i.e., regions of red and green signal colocalization), while autolysosomes exhibited red fluorescent spots. Finally, at least 10 cells were randomly selected under a 100× objective lens, and each type of fluorescent spot was manually counted for quantitative analysis.

### Statistics and reproducibility

All statistical analyses were performed using SPSS 25.0. Data are presented as the mean ± standard deviation from three independent biological replicates. Differences between two groups were determined by Student’s t-test, while comparisons among three or more groups were conducted using two-way ANOVA. Differences were considered statistically significant at *P* < 0.05. Each experiment included three independent biological replicates. siRNA library was performed as one independent experiment with three technical replicates, whereas all other experiments included three independent experiments.

## Results

### Lysosomes are damaged in UVB-induced senescence cells

**Fig. 1:**
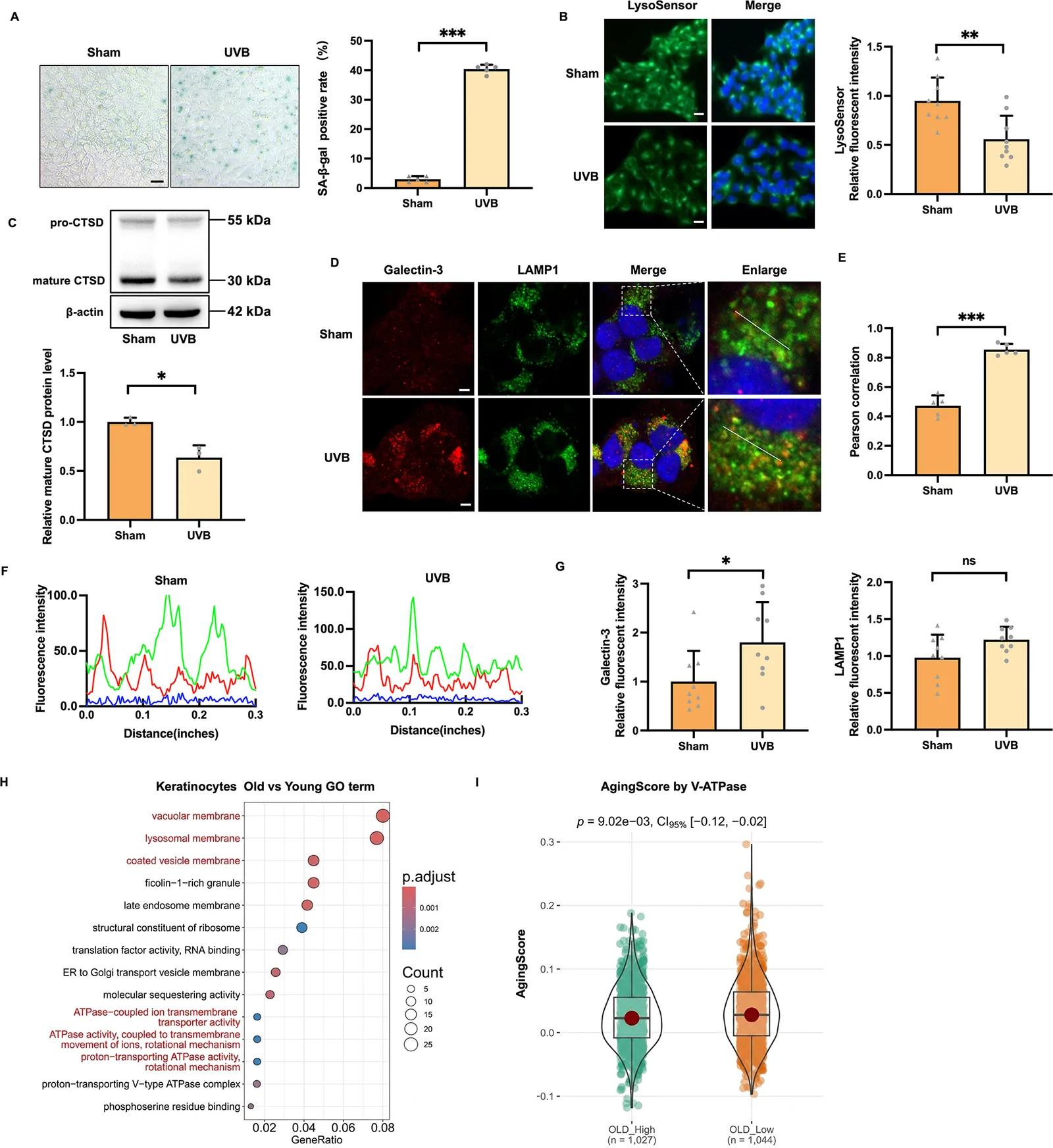
Lysosomes are damaged in UVB-induced senescent cells. (**A**) Representative images of SA-β-gal staining in HaCaT cells after UVB-induced senescence. Scale bar = 25 μm. Right panel showed the mean SA-β-gal positivity rate (*n* = 5). (**B**) Changes in LysoSensor staining in HaCaT cells after UVB-induced senescence. Scale bar = 20 μm. Right is the quantitative results of LysoSensor fluorescence intensity. (**C**) Western blot and grayscale analysis of CTSD in HaCaT cells after UVB-induced senescence. Grayscale normalized to β-actin of sham cells. (**D**) IF detection of Galectin-3 and LAMP1 expression levels in HaCaT cells after UVB-induced senescence. Scale bar = 5 μm. **E**, **F**. The colocalization analysis and line-scan intensity profiles of Galectin-3 and LAMP1 of Fig. 1D. (**G**) Quantitative results for the fluorescence intensity of Galectin-3 and LAMP1 in Fig. 1D. (**H**) GO analysis of aging and young skin in GSE130973. Higher GeneRatio indicates greater pathway relevance. (**I**) Correlation analysis of V-ATPase complex expression and aging score in keratinocytes. Data are expressed as mean ± standard deviation (SD). Student’s t-test. ns indicates no statistical significance. * *P* ≤ 0.05, ** *P* ≤ 0.01, *** *P* ≤ 0.001

Repeated UVB irradiation at 20 J/m^2^ per exposure induced a senescent phenotype in HaCaT cells, as evidenced by SA-β-gal staining, with approximately 40% of cells testing positive for SA-β-gal (Fig. 1A). Using the LysoSensor probe, a pH-sensitive fluorescent dye, we observed the fluorescence intensity was markedly reduced in senescence cells, indicating a disruption of lysosomal acidic environment (Fig. 1B). CTSD is a key lysosomal protease that is cleaved and activated within lysosomes and processed from a precursor into its mature form. Western blot analysis revealed that UVB-induced senescent HaCaT cells exhibited reduced levels of the mature form of CTSD, indicative of impaired lysosomal degradation ability (Fig. 1C). Consistently, a marked accumulation of Galectin-3 puncta colocalizing with the lysosomal marker LAMP1 was observed in UVB-induced senescence cells, reflecting lysosomal membrane permeabilization and damage (Fig. 1D, E, F and G). Additionally, we analyzed the transcriptome of keratinocytes from the single-cell RNA sequencing dataset of aged skin (GSE130973). The GO enrichment analysis showed that differentially expressed genes were significantly enriched in pathways related to the lysosomal V-ATPase complex and its function (Fig. 1H). Notably, the aging score based on the aging gene set indicates that the expression of V-ATPase-related genes is negatively correlated with the expression of aging-associated genes (Fig. 1I). These data collectively demonstrate that UVB-induced cellular senescence is accompanied by significant impairment of lysosomal integrity and function.

### ATP6V1A is a key gene involving in UVB-induced senescence

**Fig. 2:**
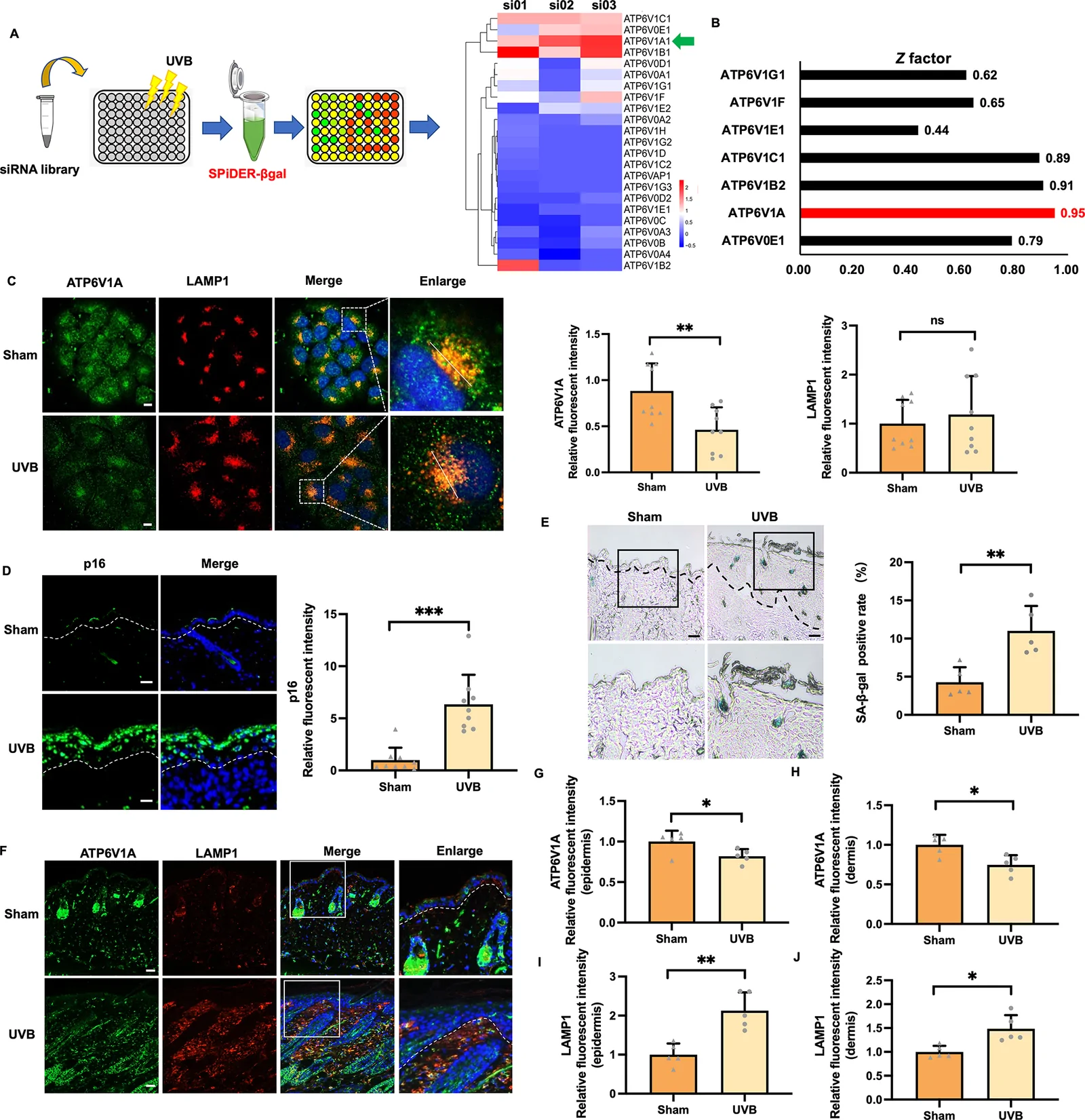
ATP6V1A expression decreases following UVB-induced senescence. (**A**) Schematic of siRNA libraries screening combined with SPiDER-βgal detection and the heat map of the results. (**B**) *Z*-score for the 7 genes with the most significant upregulation in screening. (**C**) IF was used to observe the expression of ATP6V1A and LAMP1 in UVB-induced senescent HaCaT cells. Scale bar = 10 μm. Right are the quantitative results for the fluorescence intensity of ATP6V1A and LAMP1. (**D**) IF analysis of p16 expression in the dorsal skin of mice following UVB-induced senescence. Scale bar = 12.5 μm. Right is the quantitative results of p16 fluorescence intensity. (**E**) Verification of the UVB-induced senescent mice epidermis using SA-β-gal. Scale bar = 50 μm. The right panel shows the mean SA-β-gal positivity rate (*n* = 5). (**F**) IF analysis of ATP6V1A and LAMP1 expression in the epidermis and dermis of the dorsal skin of UVB-induced senescent mice. Scale bar = 25 μm. **G**, **H**. The quantitative results for the fluorescence intensity of ATP6V1A in the epidermis and dermis of Fig. 2F. **I**, **J**. The quantitative results for the fluorescence intensity of LAMP1 in the epidermis and dermis of Fig. 2F. Data are expressed as mean ± standard deviation (SD). Student’s t-test. ns indicates no statistical significance. * *P* ≤ 0.05, ** *P* ≤ 0.01, *** *P* ≤ 0.001

To identify the V-ATPase subunits critical for UVB-induced cellular senescence, we generated an siRNA library targeting each subunit, with three independent sequences per gene, and performed a systematic screen using SPiDER-βgal activity as the readout (Fig. 2A). We found that knockdown of the V-ATPase subunits ATP6V1C, ATP6V1E, ATP6V1A, ATP6V1B1, and ATP6V1B2 significantly increased SPiDER-βgal fluorescence intensity in senescent HaCaT cells, and knockdown of ATP6V1G1 and ATP6V1F also led to a slight increase. These findings suggest that individual knockdown these genes accelerates the senescence process in HaCaT cells. *Z*-score normalization was further applied to the fluorescence intensity data from the siRNA library to facilitate standardized screening and comparative analysis, thereby establishing the relative priority of the above-mentioned 7 genes as potential contributors to senescence. Among them, ATP6V1A exhibited a *Z*-score of 0.95, indicating its critical role in the senescence process (Fig. 2B). To validate our findings, we conducted IF analysis to observe the expression and localization of ATP6V1A in senescent HaCaT cells. As expected, ATP6V1A exhibited colocalization with the lysosomal membrane protein LAMP1, and its expression was significantly reduced after UVB irradiation (Fig. 2C). To extend these observations to an in vivo setting, we established a UVB-induced mouse skin aging model, as evidenced by elevated p16 expression and positive SA-β-gal staining (Fig. 2D and E). Consistently, IF analysis showed a marked reduction in ATP6V1A fluorescence intensity in both the epidermis and dermis of UVB-irradiated mice compared with sham groups (Fig. 2F, G and H). LAMP1 expression was increased in the dermis but was no significant change in the epidermis after UVB irradiation, compared with the sham group (Fig. 2F, I and J). Collectively, ATP6V1A is involved in UVB-induced senescence process.

### ATP6V1A alleviates UVB-induced senescence in keratinocytes

**Fig. 3:**
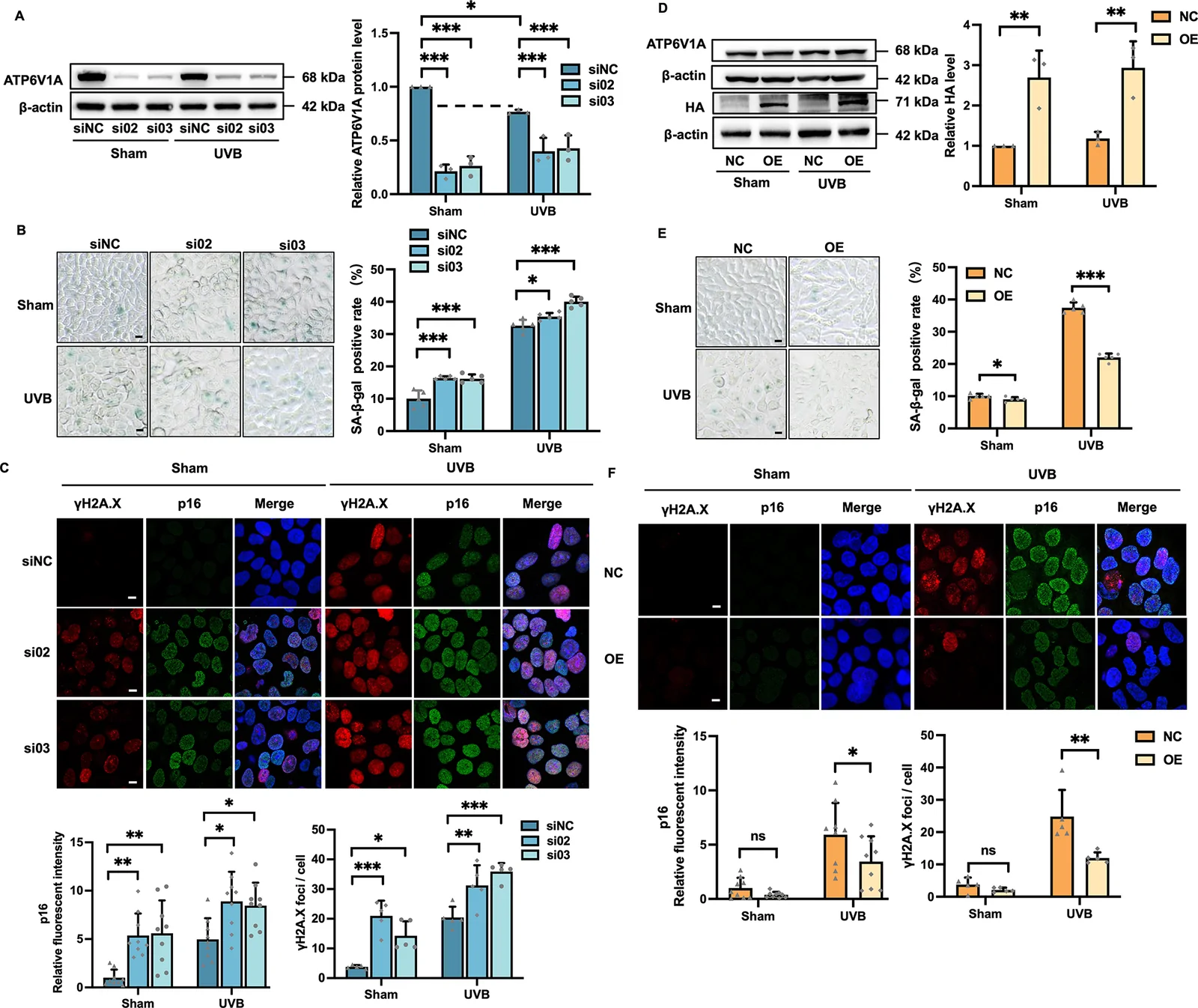
ATP6V1A regulates the UVB-induced senescence process in keratinocytes. (**A**) Western blot and grayscale analysis were used to observe the effects of ATP6V1A knockdown in HaCaT cells. Grayscale normalized to β-actin of siNC cells. (**B**) Representative SA-β-gal staining images of HaCaT cells following UVB-induced senescence after ATP6V1A knockdown. Scale bar = 25 μm. The right panel shows the mean SA-β-gal positivity rate (*n* = 5). (**C**) Representative images of p16 and γH2A.X expression in HaCaT cells undergoing UVB-induced senescence following ATP6V1A knockdown. Scale bar = 10 μm. Below are the quantification results for p16 fluorescence and γH2A.X foci. (**D**) Western Blot and grayscale analysis were used to observe the effects of ATP6V1A overexpression with HA tag in HaCaT cells. Grayscale normalized to β-actin of NC cells. (**E**) Representative SA-β-gal staining images of HaCaT cells following UVB-induced senescence after ATP6V1A overexpression. Scale bar = 25 μm. Right panel showed the mean SA-β-gal positivity rate (*n* = 5). (**F**) Representative images of p16 and γH2A.X expression in HaCaT cells undergoing UVB-induced senescence following ATP6V1A overexpression. Scale bar = 10 μm. Below are the quantification results for p16 fluorescence and γH2A.X foci. Data are expressed as mean ± standard deviation (SD). two-way ANOVA analysis. ns indicates no statistical significance. * *P* ≤ 0.05, ** *P* ≤ 0.01, *** *P* ≤ 0.001

To verify the role of ATP6V1A in UVB-induced senescence, we first used targeted siRNA to knockdown ATP6V1A expression (Fig. 3A). Under both basal conditions and UVB-induced senescence conditions, downregulation of ATP6V1A increased the SA-β-gal positivity rate in HaCaT cells compared with the control group (Fig. 3B). ATP6V1A inhibition also promoted the senescence marker, p16 expression with or without UVB exposure. In addition, DNA damage was increased in ATP6V1A downregulation, as evidenced by the accumulation of γH2A.X (Fig. 3C). We overexpressed ATP6V1A in HaCaT cells using an HA-tag expression plasmid and the overexpression of ATP6V1A was confirmed by western blot (Fig. 3D). ATP6V1A overexpression in senescent HaCaT cells significantly reduced the SA-β-gal positivity rate (Fig. 3E). Consistently, the senescence marker, p16 expression and DNA damage were both markedly alleviated in UVB-exposed group (Fig. 3F). Together, these findings demonstrate that ATP6V1A mitigates UVB-induced senescence in HaCaT cells.

### ATP6V1A enhances lysosomal function in senescent cells

**Fig. 4:**
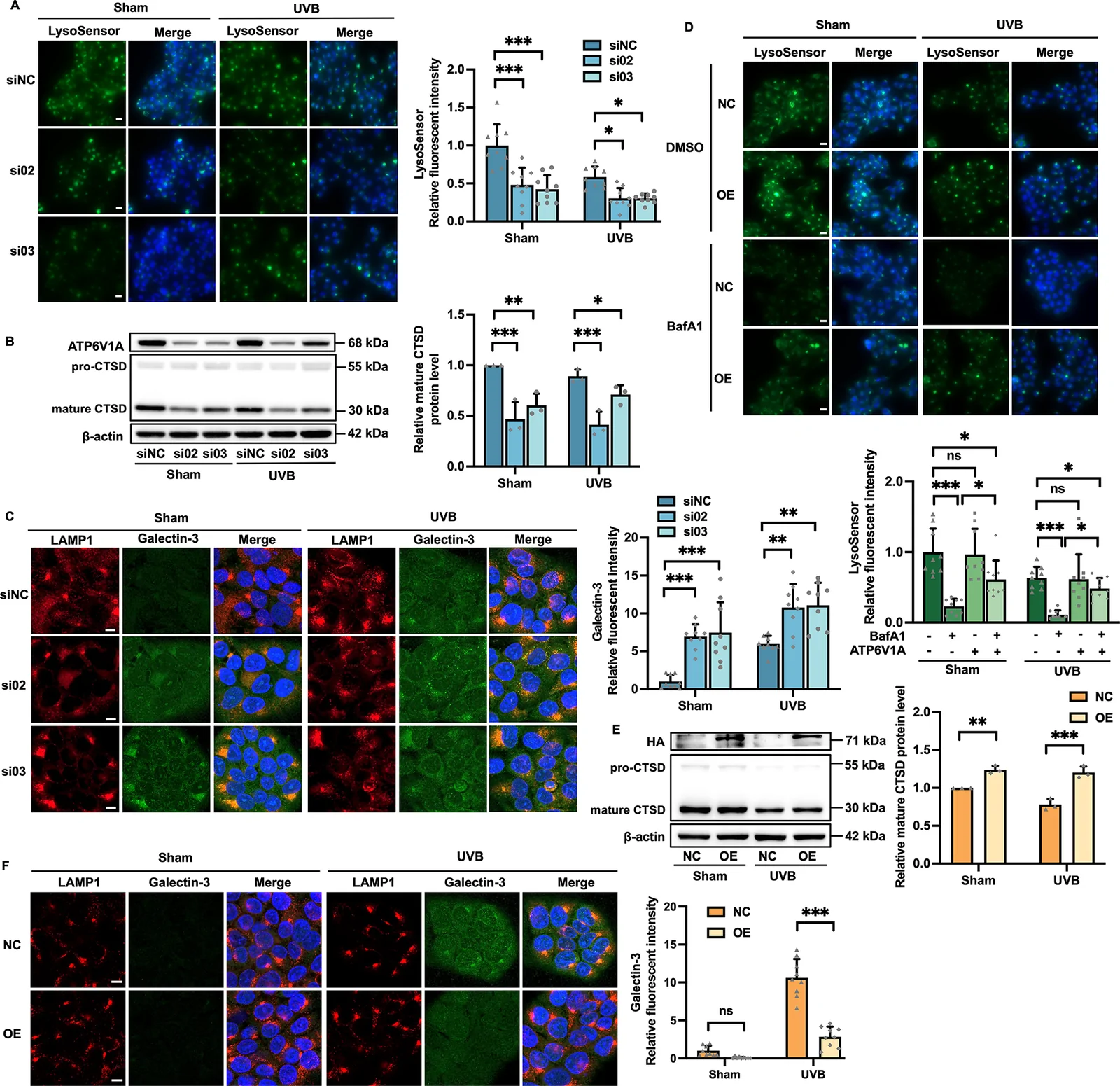
Overexpression of ATP6V1A restores lysosomal dysfunction caused by UVB-induced senescence. (**A**) LysoSensor staining in UVB-induced senescent HaCaT cells. Scale bar = 20 μm. Right is the quantitative results of LysoSensor fluorescence intensity. (**B**) Western blot analysis of mature CTSD expression in UVB-induced senescent HaCaT cells. Grayscale normalized to β-actin of siNC cells. (**C**) Representative images of LAMP1 and Galectin-3 expression in UVB-induced senescent HaCaT cells. Scale bar = 10 μm. Right is the quantitative results for the fluorescence intensity of Galectin-3. (**D**) LysoSensor detects changes in lysosomal acidity in HaCaT cells following 3 h of treatment with 100 nM BafA1, followed by overexpression of ATP6V1A and induction of UVB-induced senescence. Scale bar = 20 μm. Below is the quantitative results of LysoSensor fluorescence intensity. (**E**) Western blot analysis of mature CTSD expression in HaCaT cells with UVB-induced senescence after ATP6V1A overexpression. Grayscale normalized to β-actin of NC cells. (**F**) IF detection of LAMP1 and Galectin-3 expression in ATP6V1A-overexpressing HaCaT cells following UVB-induced senescence. Scale bar = 10 μm. Right is the quantitative results for the fluorescence intensity of Galectin-3. Data are presented as mean ± SD. two-way ANOVA. ns indicates no statistical significance. * *P* ≤ 0.05, ** *P* ≤ 0.01, *** *P* ≤ 0.001

Given that ATP6V1A is localized to lysosomes and as a subunit of V-ATPase complex, we hypothesize that its regulation of senescence depends on lysosomal function. Therefore, we first assessed the lysosomal function in HaCaT cells with knockdown of ATP6V1A. In HaCaT cells, both basal conditions and UVB-induced senescence conditions, ATP6V1A knockdown led to a significant reduction in LysoSensor fluorescence, indicating compromised lysosomal acidification (Fig. 4A). Moreover, a significant decrease in the mature form of CTSD was observed in HaCaT cells with ATP6V1A downregulation, reflecting impaired lysosomal degradative capacity (Fig. 4B). IF also revealed an exacerbated lysosomal membrane damage in ATP6V1A downregulation, as evidenced by accumulation of Galectin-3 puncta on lysosomes (Fig. 4C). As a specific inhibitor of V-ATPase, BafA1 significantly impaired lysosomal acidification in UVB-induced senescent HaCaT cells and this defect was effectively rescued by ATP6V1A overexpression (Fig. 4D). Consistently, ATP6V1A overexpression increased the levels of mature CTSD and reduced the accumulation of Galectin-3 puncta in lysosomes, suggesting restoration of lysosomal function (Fig. 4E and F). Thus, ATP6V1A maintains the acidity and membrane integrity of lysosomes in senescent HaCaT cells.

### ATP6V1A alleviates cellular senescence relying on autophagy promotion

**Fig. 5:**
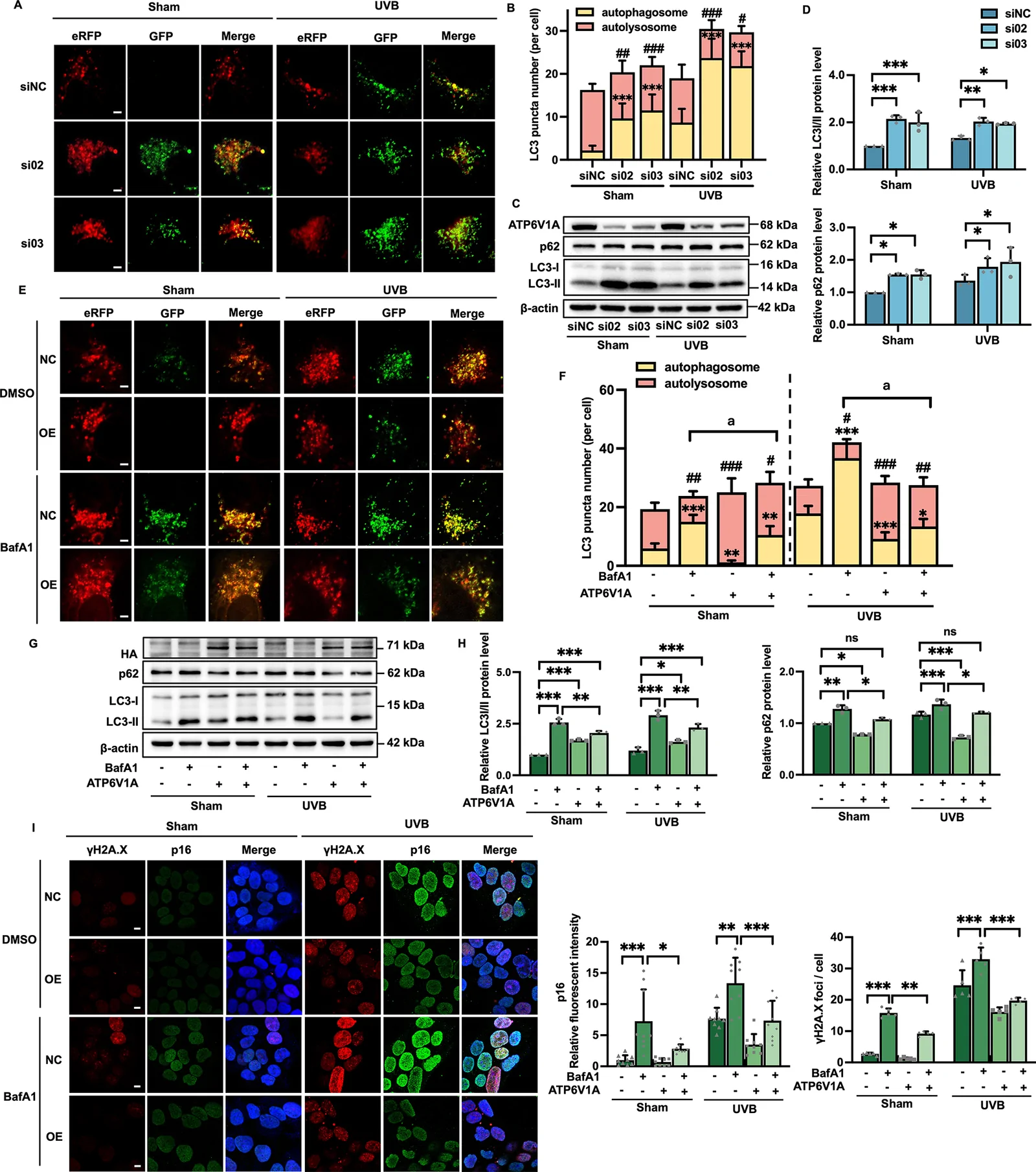
ATP6V1A alleviates UVB-induced senescence by promoting autophagy. (**A**) Representative images of UVB-induced senescent HaCaT cells with eRFP-EGFP-LC3 reporter after ATP6V1A knockdown. Scale bar = 2.5 μm. (**B**) Counting of autophagosomes and autolysosomes in Fig. 5A (*: vs. the siNC of autophagosomes; #: vs. the siNC of autolysosomes) (**C**) Western blot analysis of p62 and LC3 expression in UVB-induced senescent HaCaT cells treated with 100 nM BafA1. (**D**) Grayscale analysis of Fig. 5C and normalized to β-actin of siNC cells. (**E**) Representative images of UVB-induced senescent HaCaT cells with eRFP-EGFP-LC3 reporter. Scale bar = 2.5 μm. (**F**) Counting of autophagosomes and autolysosomes in Fig. 5E (*: vs. the siNC of autophagosomes; #: vs. the siNC of autolysosomes; a: the BafA1 vs. BafA1 combined with ATP6V1A overexpression of autophagosomes) (**G**) Western blot analysis of p62 and LC3 expression in UVB-induced senescent HaCaT cells treated with 100 nM BafA1. (**H**) Grayscale analysis of Fig. 5G and normalized to β-actin of the control groups. (**I**) Representative images of p16 and γH2A.X expression in UVB-induced senescent HaCaT cells. Right are the quantification results for p16 fluorescence and γH2A.X foci. Scale bar = 10 μm. Data are presented as mean ± SD. two-way ANOVA. ns indicates no statistical significance. * *P* ≤ 0.05, ** *P* ≤ 0.01, *** *P* ≤ 0.001. ^#^*P* ≤ 0.05, ^##^*P* ≤ 0.01, ^###^*P* ≤ 0.001. a: *P* ≤ 0.001

Given the critical role of lysosome in sustaining autophagy activity and flux, we hypothesize that ATP6V1A regulates cellular senescence through autophagy. Autophagy flux was quantified in HaCaT cells using the eRFP-EGFP-LC3 reporter system. Compared with non-irradiated cells, senescent cells exhibited increased yellow puncta, indicative of autophagosome accumulation, which was further aggravated by ATP6V1A knockdown (Fig. 5A and B). Consistent with this, LC3 and p62 levels were elevated, suggesting impaired lysosomal degradation of autophagosomes in senescent cells (Fig. 5C and D). Moreover, ATP6V1A overexpression reduced yellow puncta and increased red puncta, indicating enhanced autophagic flux in senescent cells, as further supported by reduced LC3 and p62 expression (Fig. 5E, F, G and H). BafA1 was used to impair lysosomal function, resulting in autophagosome accumulation and elevated both LC3 and p62 levels in senescent cells, all of which were reversed by ATP6V1A overexpression (Fig. 5E, F, G and H). Therefore, BafA1 aggravated UVB-induced cellular senescence, as evidenced by increased γH2A.X and p16 expression, and this phenotype was markedly attenuated by ATP6V1A overexpression (Fig. 5I). Collectively, these results suggest that ATP6V1A alleviates UVB-induced cellular senescence by restoring lysosomal function and promoting autophagic flux.

## Discussion

Lysosomal dysfunction is the pathological basis of cellular senescence [18]. Existing research indicates that lysosomal dysfunction is a key factor in the onset and progression of neurodegenerative diseases [19]. In the context of bleomycin-induced cellular senescence, oxidative stress accumulation is likely to compromise lysosomal integrity by increasing lysosomal membrane permeability [20, 21]. As a sensor of lysosomal membrane damage, Galectin-3 may serve as an indicator of lysosomal instability and aging-associated cellular stress. Previous studies have shown that Galectin-3 is upregulated in various aging-related diseases. Serum Galectin-3 levels in patients with Parkinson’s disease have been reported to increase significantly with age. Such aberrant elevation may reflect lysosomal dysfunction and impaired lysosome-dependent degradation, thereby contributing to the accumulation of senescence-related substrates and the persistence of cellular senescence [22, 23]. Consistent with these findings, our study showed that UVB-induced senescent HaCaT cells exhibited reduced lysosomal acidity and Galectin-3 upregulation, suggesting increased lysosomal membrane permeability and impaired degradative capacity. In addition, Galectin-3 has been reported to be highly secreted by senescent mesenchymal stem cells and may function as a senescence-associated microenvironmental mediator that promotes neighboring colorectal cancer cell proliferation through ERK1/2-MAPK signaling [24]. However, the relationship between Galectin-3 and cellular senescence appears to be context-dependent. Kim et al. reported that Galectin-3 depletion was sufficient to induce premature senescence through p27^KIP1^ accumulation, suggesting that Galectin-3 may suppress senescence in certain proliferative cell contexts [25]. Therefore, Galectin-3 upregulation during aging-related stress may not merely represent a direct driver of senescence or a passive marker of impaired lysosome-dependent degradation, but may also reflect a compensatory or context-specific response to cellular damage, lysosomal instability, and tissue remodeling.

Intact V-ATPase function is essential for normal lysosomal physiological activity [26–28]. As a core structural subunit of V-ATPase, ATP6V1A is a key molecule for maintaining normal lysosomal function. Studies have shown that downregulation of ATP6V1A disrupts the acidic environment of lysosomes [29, 30]. In head and neck squamous cell carcinoma, the accumulation of hypoxia-inducible factor-1α under hypoxic conditions directly downregulates ATP6V1A, leading to impaired lysosomal degradation function [31]. Concurrently, ATP6V1A activates the ataxia-telangiectasia mutation pathway, promoting the progression of telangiectasia by inhibiting lysosomal function [32]. In senescent cells, we observed decreased ATP6V1A expression and found that downregulation of ATP6V1A promotes UVB-induced senescence and exacerbates UVB-induced impairment of lysosomal function, suggesting that ATP6V1A may also play a role in the UVB-induced cellular senescence process by regulating lysosomal function. In addition to regulating lysosomal function, V-ATPase is closely associated with the maintenance of skin homeostasis, and its functional abnormalities directly affect the normal physiological state of the skin. Studies have shown that mutations in the ATP6V0A2 gene lead to wrinkled skin syndrome, characterized by impaired glycosylation and abnormal cortical neuron migration [33]. Mutations in the ATP6V1E1 and ATP6V1A genes, on the other hand, lead to severe facial skin laxity and fat redistribution [34]. However, to date, there have been few reports on the role and mechanisms of ATP6V1A in skin senescence, particularly in the process of UVB-induced senescence.

As a key cellular degradation mechanism, autophagy plays a role in clearing dysfunctional organelles and metabolic byproducts [35]. It is currently widely accepted that inhibiting autophagy promotes aging by affecting the autophagy-lysosomal system [36]. When autophagy fails to function properly, toxic proteins such as misfolded proteins and damaged mitochondria accumulate in the body, promoting the aging process [37]. Autophagy plays a role in the aging process across various organs. Studies have shown that inhibiting autophagy in neurons leads to the development of neurodegenerative diseases [38]. Conversely, significantly reduced autophagy levels have been observed in aging bone marrow mesenchymal stem cells, and restoring autophagy can partially reverse the aging process [39]. Here, we found that knocking down ATP6V1A in HaCaT cell leads to impaired autophagy. Combined with the aging phenotype observed upon ATP6V1A knockdown, this suggests that ATP6V1A may regulate UVB-induced senescence by modulating autophagy, consistent with previous studies on ATP6V1A and autophagy [40].

As an autophagy inhibitor, BafA1 blocks lysosomal acidification by inhibiting V-ATPase function, thereby affecting the fusion of autophagosomes with lysosomes [41, 42]. Recent studies have shown that BafA1 treatment can induce cellular senescence by inhibiting autophagy [36, 43]. Consistent with our results, BafA1 treatment induced senescence in HaCaT cells, regardless of whether UVB was induced, as evidenced by increased expression of p16 and γH2A.X. However, overexpression of ATP6V1A in BafA1-treated HaCaT cells reversed the BafA1-induced inhibition of autophagy and reduced the expression of these markers. This phenomenon is likely due to the restoration of V-ATPase function following ATP6V1A overexpression, which stabilizes lysosomal homeostasis and allows autophagy to proceed normally. Based on the relationship between lysosomes and autophagy, we have identified the specific mechanism by which ATP6V1A regulates senescence of the skin, thereby providing a new intervention strategy for modulating UVB-induced senescence.

In summary, this study identified ATP6V1A by analyzing single-cell data from aged skin and combining it with a V-ATPase-targeted siRNA library, and confirmed that it plays a role in the UVB-induced cellular senescence process by inhibiting autophagy through the regulation of lysosomal function. During UVB-induced cellular senescence, ATP6V1A expression decreases, inhibiting lysosomal function and consequently suppressing autophagy, which further promotes senescence. It is worth noting that some studies have reported that downregulation of ATP6V1A expression in bone tissue can delay osteoblast senescence by alleviating oxidative stress [44]. This finding contradicts the results of the present study, suggesting that the regulatory role of ATP6V1A in senescence may be tissue-specific. Therefore, future research should further investigate the functional mechanisms and downstream regulatory pathways of ATP6V1A in UVB-induced senescence, and utilize potential targets to prevent and delay it.

## Conclusion

In summary, we identified ATP6V1A as a key factor in senescence through single-cell transcriptomic from aged skin tissue integrated V-ATPase-targeted siRNA library screening. We found that ATP6V1A expression decreases following UVB-induced cellular senescence, leading to impaired lysosomal function, which in turn inhibits autophagy and accelerates the senescence process. Conversely, overexpression of ATP6V1A significantly delays UVB-induced cellular senescence. These findings provide new therapeutic insights for the prevention and treatment of UVB-induced cellular senescence.

## Supplementary Information

Below is the link to the electronic supplementary material.

Supplementary Material 1

## Data Availability

The dataset presented in this study is available in the GEO database. The accession number is GSE130973, and it can be accessed via the following URL: https://www.ncbi.nlm.nih.gov/geo/query/acc.cgi?acc=GSE130973.
