# Supplementary material for: V-ATPase-targeted siRNA library screening reveals ATP6V1A negatively regulates UVB-induced keratinocyte senescence
Source: Biol Direct. 2026 Jul 15;21:195. doi: 10.1186/s13062-026-00891-4 (PMC13656529; doi:10.1186/s13062-026-00891-4)

Uncropped Gels and Blots in Figure1

Figure1 B

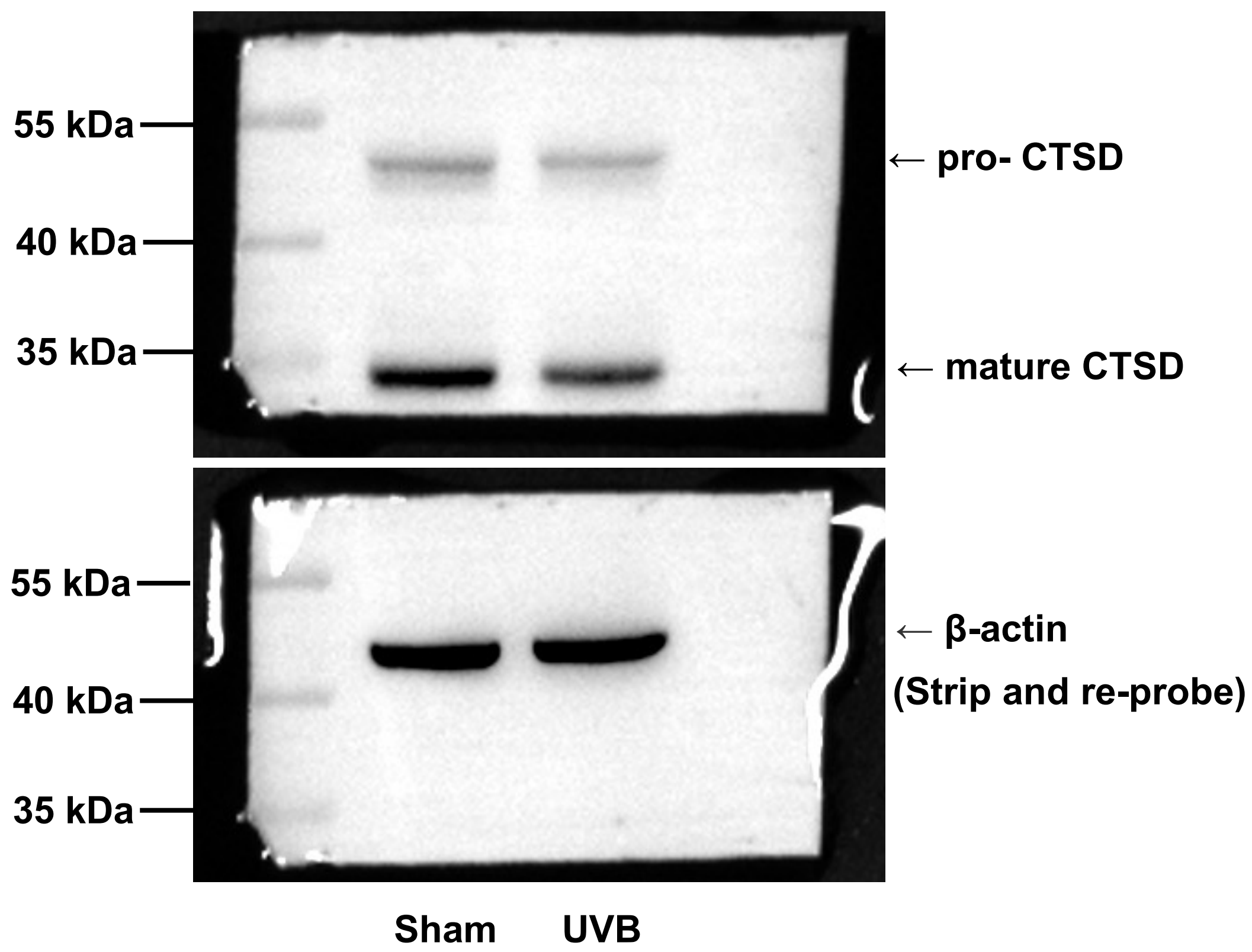

Uncropped Gels and Blots in Figure3

Figure3 A

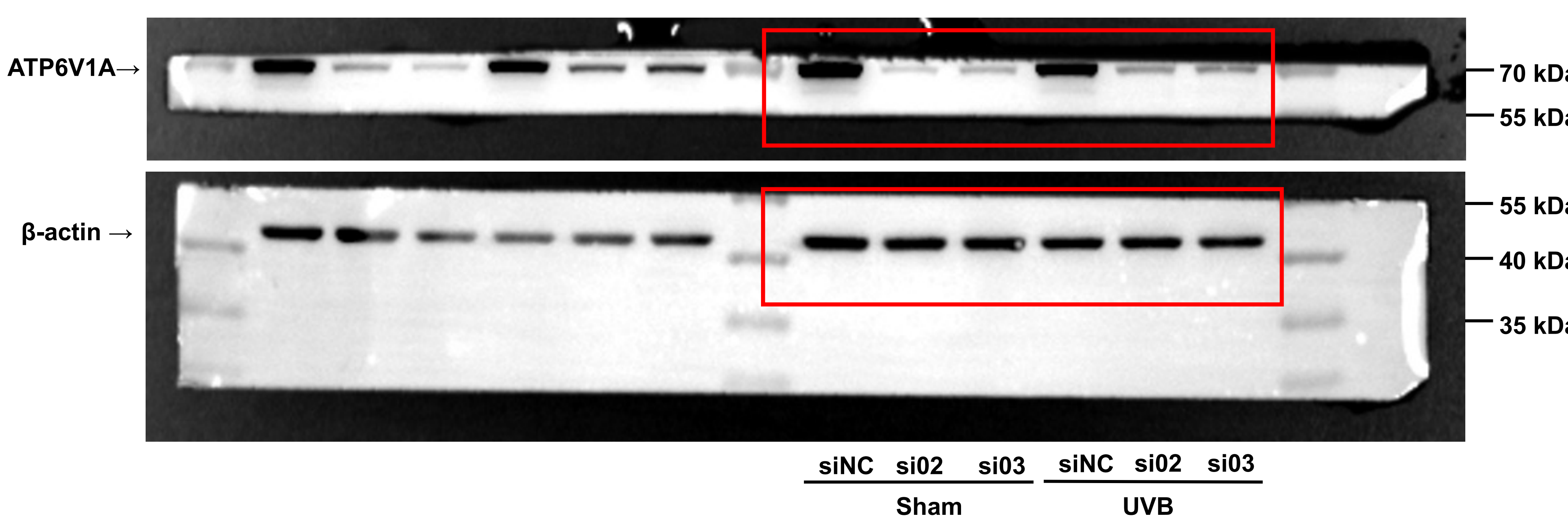

Figure3 D

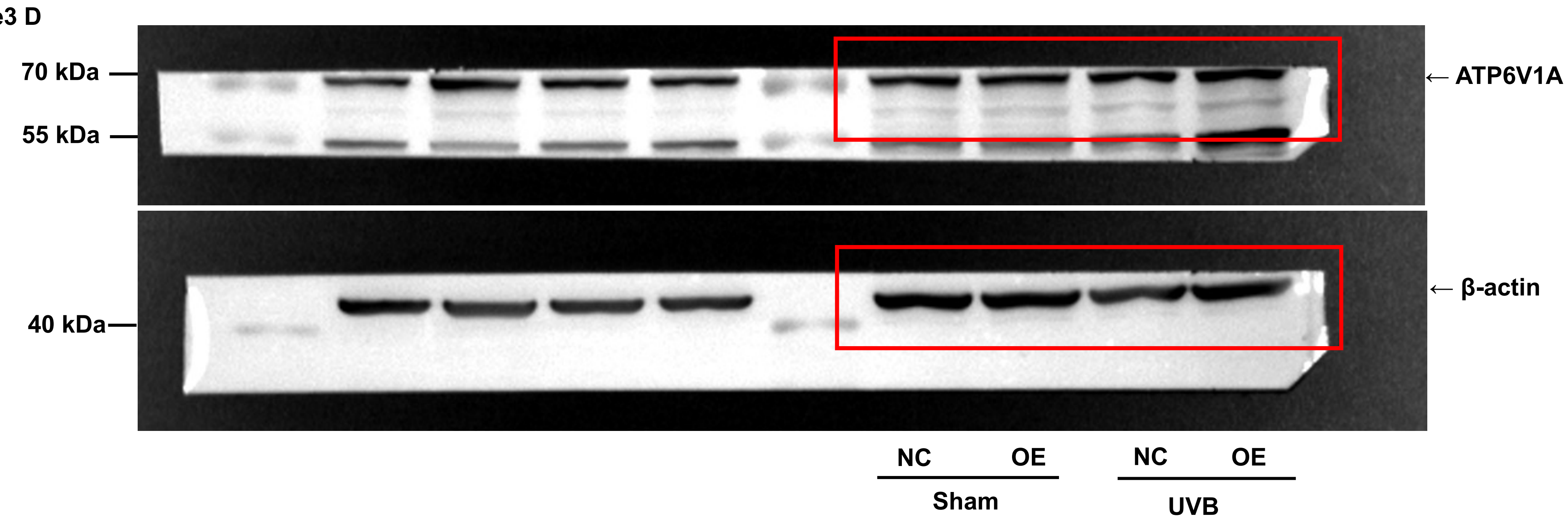

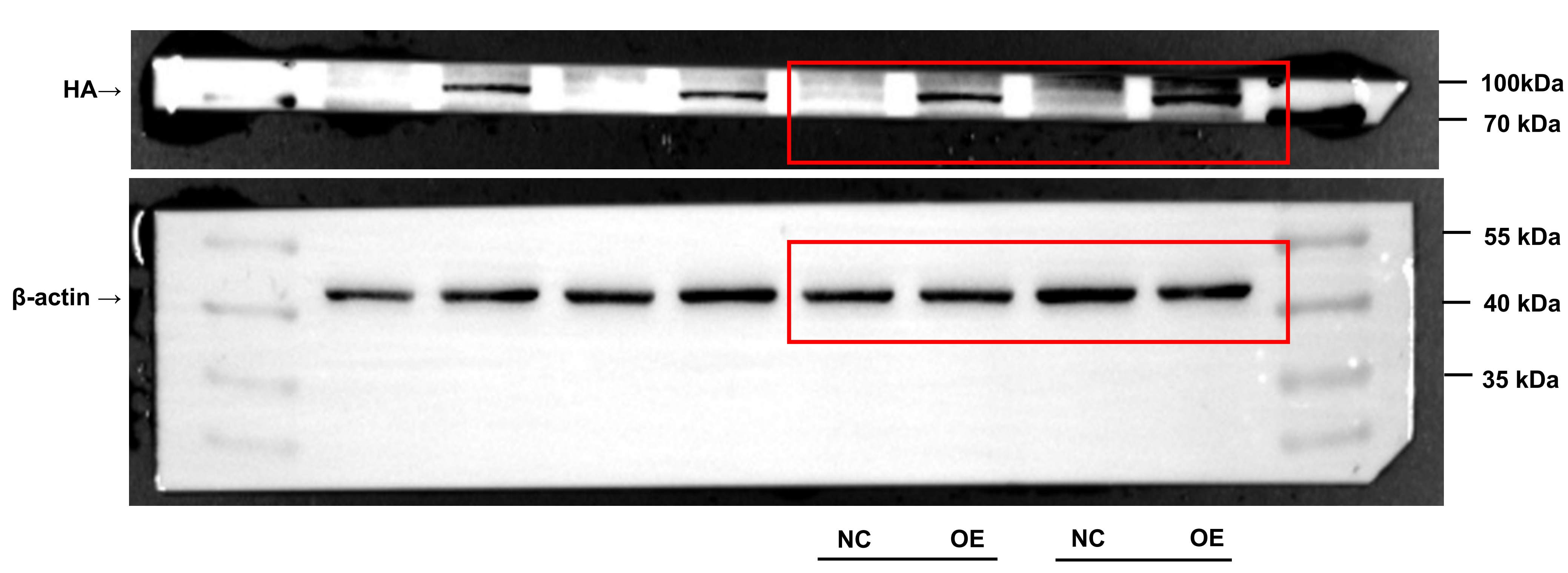

Uncropped Gels and Blots in Figure4

Figure4 A

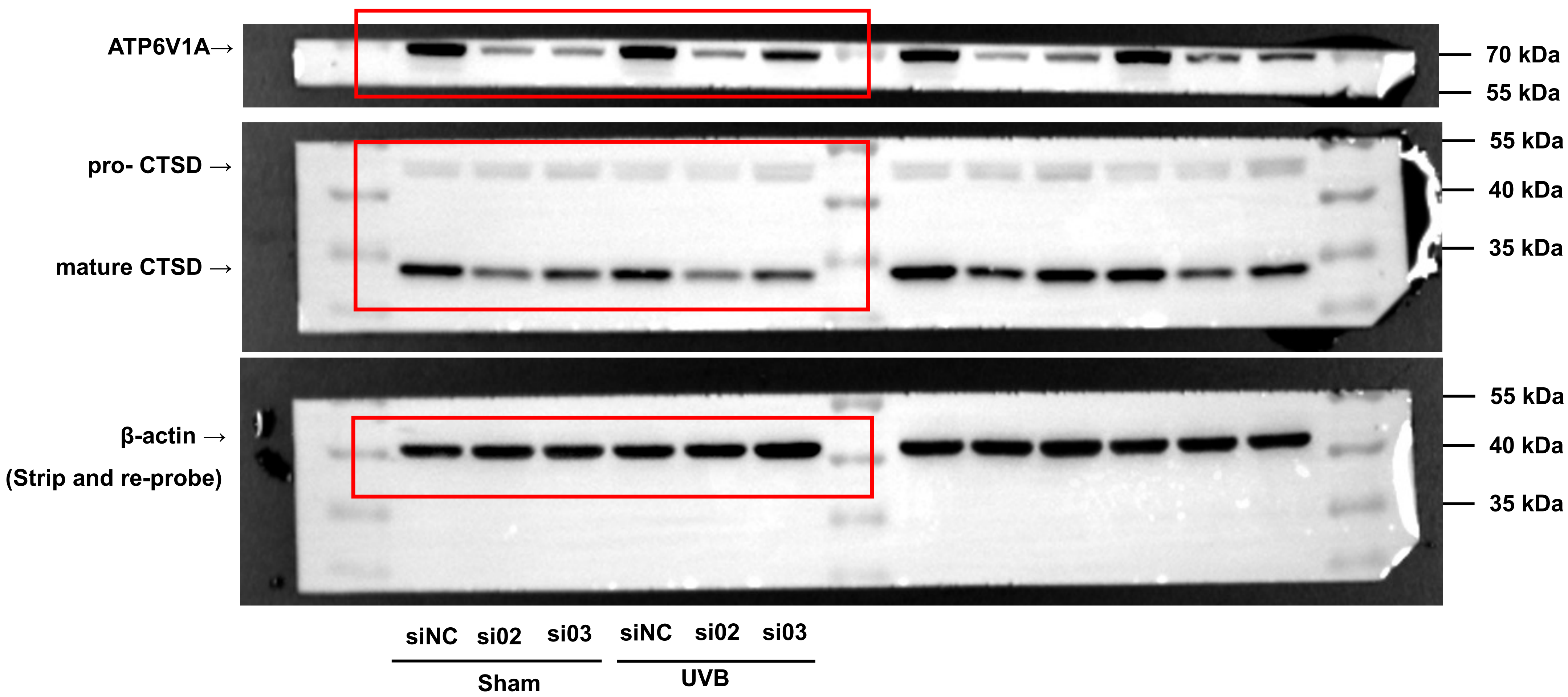

Figure4 E

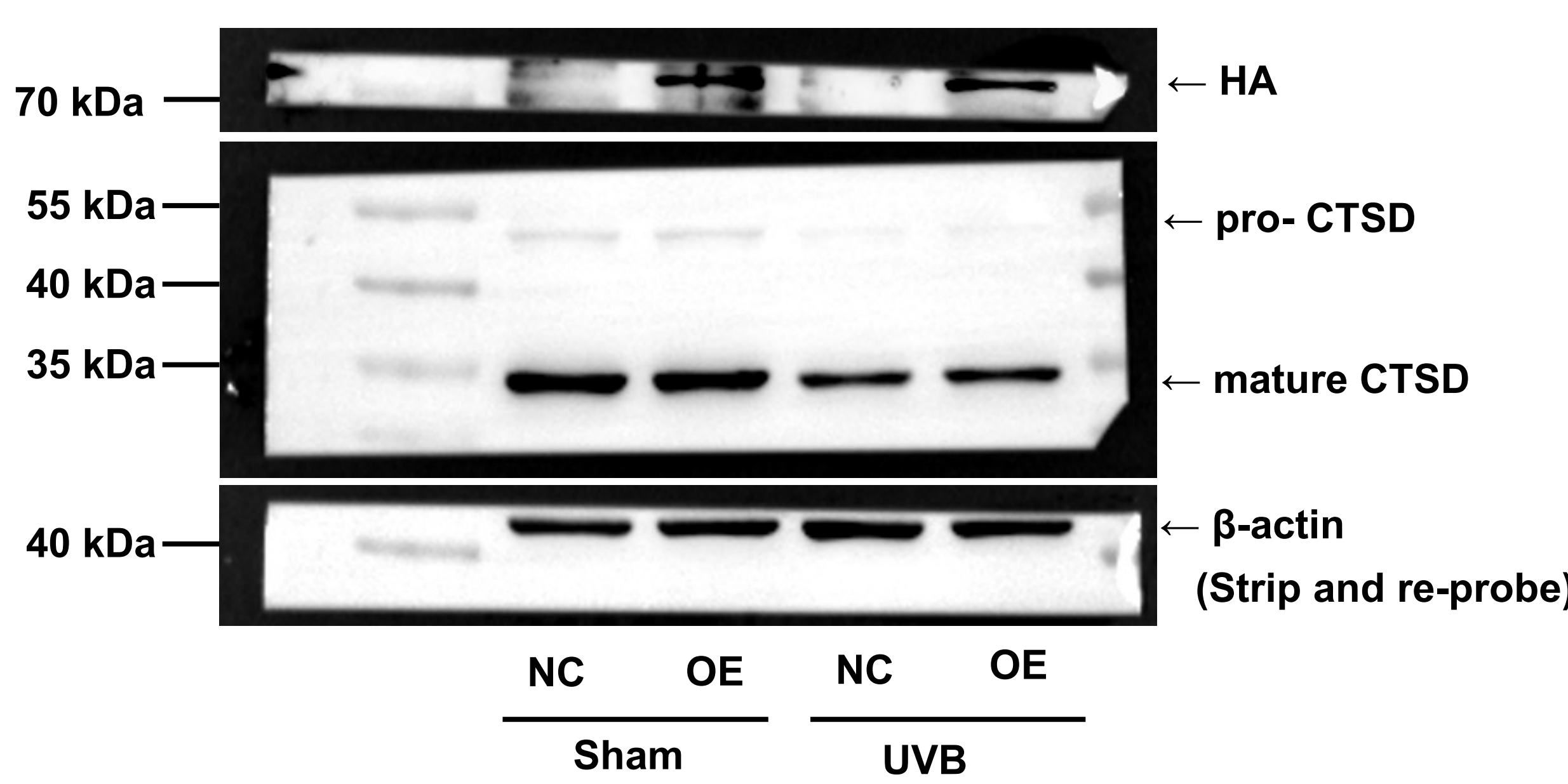

Uncropped Gels and Blots in Figure5

Figure5 C

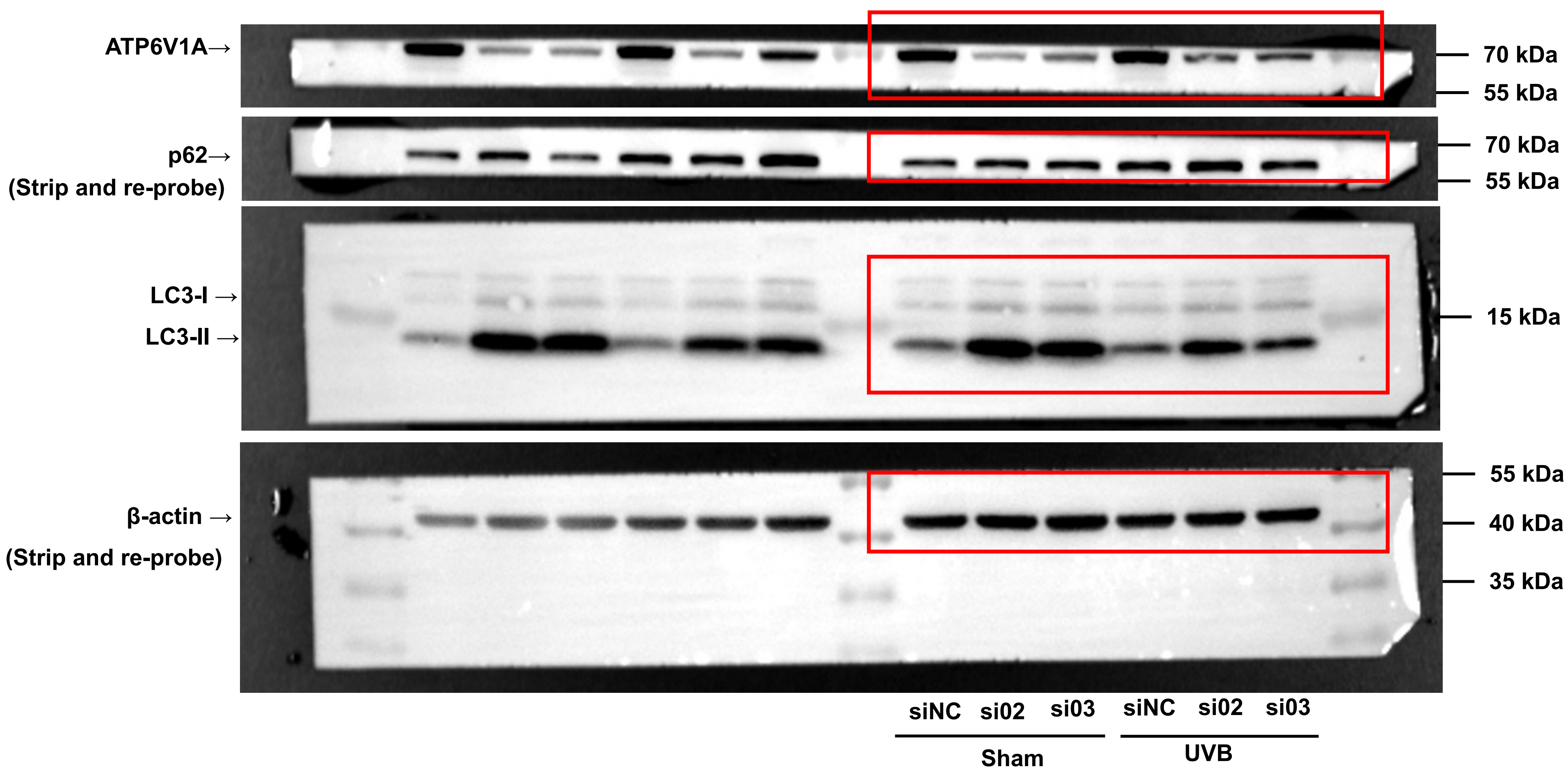

Figure5 G

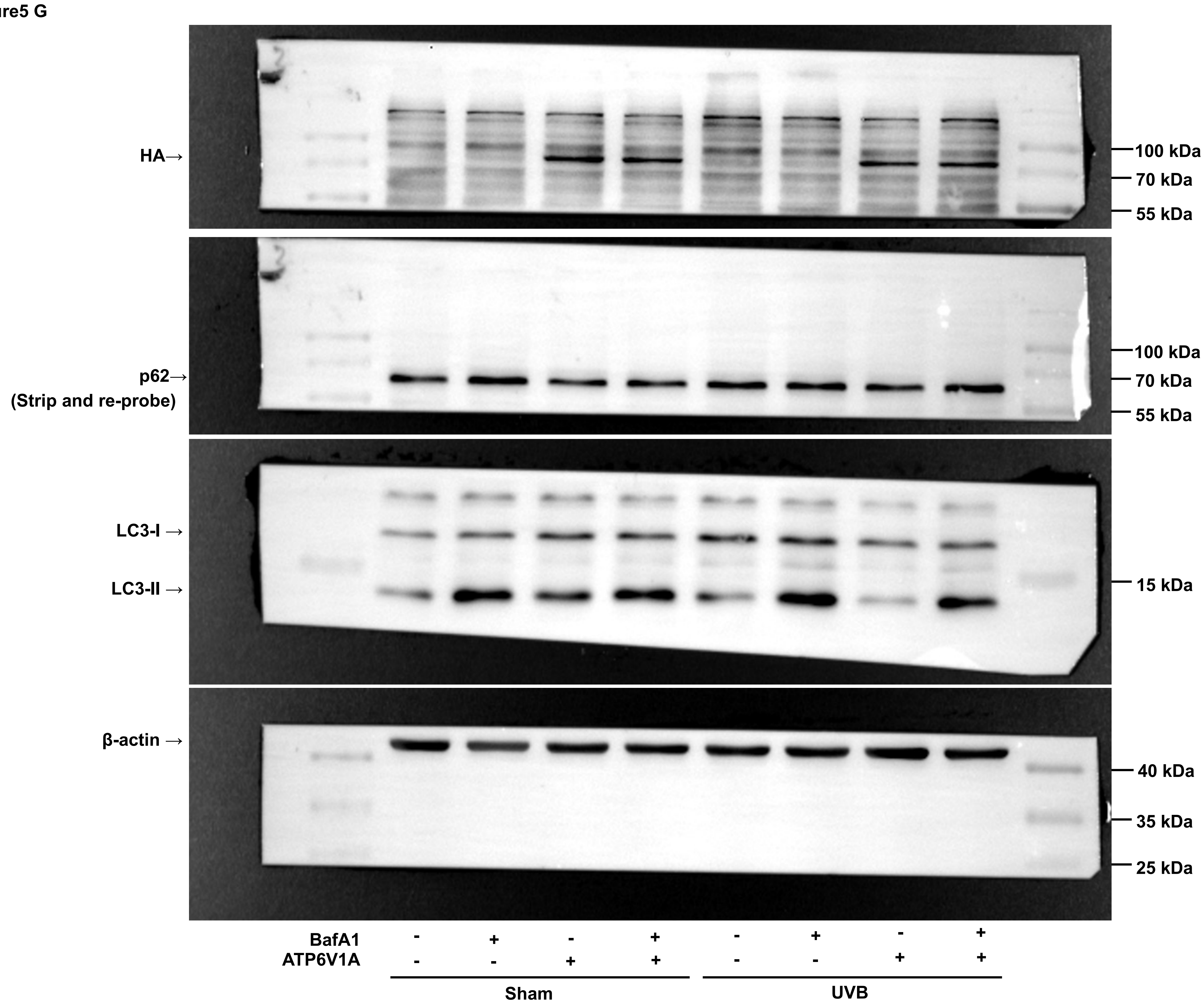

Supplement: Supplementary file 1 [file 13062_2026_891_MOESM1_ESM.pdf]
